# Temptation at Work

**DOI:** 10.1371/journal.pone.0053713

**Published:** 2013-01-30

**Authors:** Alessandro Bucciol, Daniel Houser, Marco Piovesan

**Affiliations:** 1 Department of Economics, University of Verona, Verona, Italy; 2 Interdisciplinary Center for Economic Science, George Mason University, Fairfax, Virginia, United States of America; 3 Institute of Food and Resource Economics, University of Copenhagen, Copenhagen, Denmark; The University of South Wales, Australia

## Abstract

To encourage worker productivity, companies routinely adopt policies requiring employees to delay gratification. For example, offices might prohibit use of the internet for personal purposes during regular business hours. Recent work in social psychology, however, suggests that using willpower to delay gratification can negatively impact performance. We report data from an experiment where subjects in a Willpower Treatment are asked to resist the temptation to join others in watching a humorous video for 10 minutes. In relation to a baseline treatment that does not require willpower, we show that resisting this temptation detrimentally impacts economic productivity on a subsequent task.

## Introduction

The office place is filled with tempting distractions from one's work, including everything from socialization with colleagues to napping. For example, in some workplaces a temptation is the Internet. Indeed, a widely cited survey conducted in 2005 by America Online and Salary.com ranked personal Internet use as the number one way people waste time at work [1]. To encourage worker productivity, some offices adopt policies prohibiting Internet use during work hours, with some even monitoring employees' Internet activities. As a result, many employees delay gratification and wait until the workday ends to use the Internet. However, a well-established result from social psychology is that using willpower to delay gratification, whether from the Internet or any of many other temptations, can detrimentally impact performance on subsequent tasks [2].

One reason that resisting temptations can have adverse impact on subsequent performance is that using willpower consumes an individual's energy [3]–[4]. Once this energy is depleted, willpower can become more difficult to exercise which, in turn, can have detrimental impact on one's ability to delay gratification [5]–[9]. These ideas have received increasing attention by not only psychologists but also economists [10]–[12].

Our goal is to understand whether exposure to a prohibited tempting item reduces work productivity on a subsequent task. To the best of our knowledge, the relation between temptation and labor productivity has been addressed only by Bucciol et al. [13] in a field experiment with children. That paper reports data indicating the productivity of children is reduced after they are exposed to temptation.

### The experiment

Our experiment was conducted in 3 sessions at the Laboratory for Experimental Economics (LEE) of the University of Copenhagen. Our analysis is based on 60 subjects recruited using ORSEE [14]. The experiment was programmed using the software z-tree [15]. On average, subjects spent 75 minutes in the experiment and earned 125 Danish crowns (DKK, about 22 USD). After the experiment we administered a short questionnaire about subjects' characteristics; Table 1 summarizes the information we know on the sample.

**Table 1:** Mean Participant Characteristics by Treatment.

	Whole Sample	NWT	WT	Rank-sum test
N. observations	60×13 = 780	25×13 = 325	35×13 = 455	
Payoff (DKK)	7.444	7.639	7.304	1.261
Age	25.650	26.200	25.257	2.753***
Female	0.333	0.200	0.429	−6.672***
Danish	0.567	0.600	0.543	1.587
Field: Science	0.350	0.320	0.371	−1.484
Field: Humanities	0.167	0.200	0.143	2.110**
N. household members	3.133	3.440	2.914	7.093***
Personal budget (k DKK)	3.707	4.032	3.474	1.679*

Note: the last column reports the non-parametric Wilcoxon rank-sum test statistic (also known as Mann-Whitney test), under the null hypothesis that the two independent samples (NWT and WT) are from populations with the same distribution.

* = significant at 10%;

** = significant at 5%;

*** = significant at 1%.

The experiment consists of three phases. In Phase 1, subjects perform three counting tasks; in Phase 2 they have the possibility to watch a funny video; in Phase 3 they perform ten counting tasks. Subjects in each session are randomly assigned in two treatments: No Willpower Treatment (NWT) and Willpower treatment (WT). The only difference between treatments occurs in Phase 2. In NWT the video starts automatically whereas in WT subjects just see a red button labeled “VIDEO” on their screen. The temptation is made salient by ensuring all subjects could hear the sounds of the video. Subjects are not monitored in that no experimenter is visibly present during this phase. WT subjects are aware that the video will start if they press the red button, but they are asked not to do so. If they press the red button, a text message in their screen warns that they should not have pressed the button. This is meant to recognize that button pressure might be accidental. The video appears if subjects press the button once more, but in this case they are considered overwhelmed by temptation and therefore excluded from the analysis. In the experiment we experienced just one case of pressing the red button twice.

In phases 1 and 3 we measure subjects' productivity through the counting tasks. In each task subjects watch a video where 8 individuals are passing each other one or more balls of different colors. Subjects have to count the exact number of times a specific ball moves from one player to another one. When the video is over, subjects have to report their answer. The level of complexity varies from task to task with the number of ball passes subjects are asked to count. At the end of each counting task they receive a feedback with the correct answer, their guess and the points earned. Points are assigned according to the precision of the answer. Subjects earn 100 points if they precisely report the correct answer, 65 points if the difference between their guess and the correct answer is 1 (either from above or below), 50 if the difference is 2 and 0 points if the difference is bigger than 2. At the end of the experiments points are converted in Danish crowns (DKK), with the conversion set at 10 points = 1 DKK. Note that an advantage to using our counting task is that participants produce answers and, in close analogy with any piece-rate economic production task, their productivity is quality (accuracy) weighted. Further details regarding the experiment, instructions and screenshots are available in the online Text S1.

**Figure 1:**
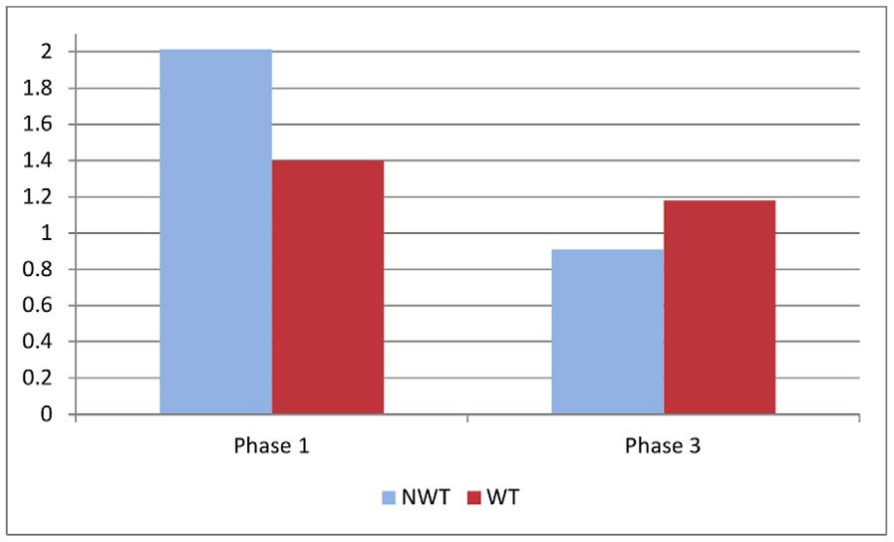
Mean Number of Mistakes.

The counting task requires concentration that is depleted (or not replenished) in the willpower treatment [16]. Figure 1 reports the average mistake (measured as the absolute difference between the correct answer and the answer reported in each task by each subject) in the NWT and WT samples, in Phase 1 and in Phase 3. The size of mistakes made in the four sub-samples is statistically different according to a two-way ANOVA interaction test comparing the two NWT and WT groups over the two phases (F(1,776) = 4.14, p = 0.04). It is also clear that mistakes occur less frequently in Phase 1 of the WT than NWT (mean mistakes are 1.40 and 2.013 in WT and NWT, respectively); the reverse is true in Phase 3 (mean mistakes are 1.18 and 0.91 in WT and NWT, respectively). It follows that imprecision (i.e., size of mistakes) on the assigned task is greater in WT than NWT after exposure to temptation. In the next section we aim to estimate the effect of temptation on productivity, with and without controlling for subjects' characteristics.

## Results

Table 2 shows the output of four regression equations where the dependent variable is the mistake, measured as the absolute difference between the correct answer and the answer reported in each task by each subject. Positive estimates of the coefficients mean that the subject is more likely to make larger mistakes. To account for the possibility that participants in WT and NWT may have different mean precision, we adopt a difference-in-difference strategy. In the specification we therefore treat three dummy variables: one indicates the treatment, to capture any between-treatment differences in “skill”, one is the Phase (3 as opposed to 1), to capture any “learning”, and one the interaction between the group and the phase, to capture the “temptation effect” of primary interest.

**Table 2:** Partial Correlates of Productivity.

	(1)	(2)	(3)	(4)
Method	Panel Poisson	Panel Poisson	Panel GLS	Panel GLS
WT	−0.363*	−3.555*	−0.613	−5.926*
	(0.214)	(1.953)	(0.470)	(3.258)
Phase 3	−0.792***	−1.499***	−1.101***	−1.805***
	(0.105)	(0.149)	(0.365)	(0.444)
WT×Phase 3	0.621***	1.076***	0.881*	1.290**
	(0.142)	(0.193)	(0.478)	(0.582)
Difficulty		2.541***		2.764***
(correct answer/video length)		(0.344)		(1.010)
Age		−0.021		−0.018
		(0.041)		(0.069)
Female		0.582*		1.016*
		(0.315)		(0.602)
Danish		−0.663		−0.316
		(0.406)		(0.621)
Field: Science		−0.248		−0.224
		(0.302)		(0.525)
Field: Humanities		−0.800**		−0.857
		(0.339)		(0.564)
N. household members		−0.220		−0.113
		(0.167)		(0.244)
Personal budget (k DKK)		0.070		0.090
		(0.061)		(0.096)
WT×Difficulty		−1.586***		−1.608
(correct answer/video length)		(0.442)		(1.323)
WT×Age		0.133**		0.242**
		(0.061)		(0.105)
WT×Female		−0.852**		−1.065
		(0.389)		(0.716)
WT×Danish		−0.043		−0.690
		(0.484)		(0.765)
WT×Field: Science		0.142		0.392
		(0.385)		(0.670)
WT×Field: Humanities		1.186***		1.873**
		(0.460)		(0.798)
WT×N. household members		0.0780		−0.152
		(0.203)		(0.314)
WT×Personal budget (k DKK)		0.054		0.137
		(0.090)		(0.148)
Constant	0.700***	0.937	2.013***	1.472
	(0.159)	(1.492)	(0.359)	(2.348)
				
Number of observations	780	780	780	780
Number of individuals	60	60	60	60
				
Wald Chi^2^ test	60.140	158.620	9.670	51.110
P-value	[0.000]	[0.000]	[0.022]	[0.000]
Hausman test	0.000	0.100	0.000	0.140
P-value	[0.999]	[0.999]	[0.999]	[0.998]

Dependent variable: absolute difference between correct and reported answer in each task. Estimation methods: (1) and (2): Panel Poisson regression with random effects; (3) and (4): Panel GLS regression with random effects. Robust standard errors in round parentheses; p-values in squared parentheses.

* = significant at 10%;

** = significant at 5%;

*** = significant at 1%.

Column (1) reports the output of a panel Poisson model with random effects and only these three variables in the specification. We find a significant effect of all the variables:

Skill effect: The group variable “WT” is negative, suggesting that the WT sample is more skilled than the NWT sample (the effect is significant at 10%).
Learning effect: The phase variable “Phase 3” is negative, suggesting that learning occurs (the effect is significant at 1%).
Temptation effect: The interaction variable “WT×Phase 3” is positive. This suggests that, on average, the WT sample is more likely to make larger mistakes than the NWT sample, after exposure to temptation in Phase 2 (the effect is significant at 1%).

As a robustness check we enrich the specification with further control variables: one for the task complexity (video difficulty, measured as the ratio between the correct answer and the video length in seconds), as well as demographic variables for age, gender, nationality, and variables for the field of studies (science or humanities, as opposed to social sciences), number of household members (apart from the subject), and personal budget. These variables are added because they can potentially influence our dependent variable (e.g., the mistake could be larger when the task is more difficult.) All the control variables are also interacted with WT to capture any between-treatment heterogeneity in participants' characteristics. In particular, this enriched specification should remove potential biases due to the different characteristics of the two treatment groups (see Table 1). Although task complexity and some characteristics of the subjects seem relevant predictors of the final outcome, our above findings are still confirmed.

The models in Columns (1) and (2) also allow us to predict the expected mistake size, as an exponential function of the specification. Predictions from Column (1) coincide with the descriptive statistics shown in Figure 1, while predictions from Column (2) differ because they take into account the characteristics of the two treatment groups. In this case we find that a subject with average characteristics will make in Phase 1 a mistake 1.93 times larger than the overall average mistake. In contrast, the same individual in Phase 3 would make just 0.43 times the average mistake if not exposed to temptation, and 1.26 times the average mistake if exposed to temptation. Thus, all else equal, mistakes subsequent to temptation exposure are nearly three times as large in the absence of temptation exposure.

Columns (3) and (4) report a panel GLS model with random effects on the same regression equations as Columns (1) and (2). The qualitative findings reported above are confirmed. Results in Table 2 are preserved also when using models with fixed effects rather than random effects.

## Discussion

In this paper we find that subjects required to resist the temptation of a humorous video made significantly larger mistakes on a subsequent counting task. This result is consistent with the standard resource depletion theoretical framework from social psychology, as discussed in the introduction. In particular, willpower depletion resulting from resisting the temptation to watch the video may have made concentration on a subsequent labor productivity task more difficult. Alternatively, watching the video may have promoted resource replenishment, enabling higher levels of concentration on the subsequent task. Both interpretations are consistent with the resource depletion theoretical framework, and thus we would expect those who were resisting watching the video to have lower subsequent productivity than those who did not need to resist this temptation [13].

Recent work [6]–[9] suggests the extent to which participants believe that willpower is a depletable resource can influence their own ego-depletion and task performance. In light of our findings, it is possible that participants believed themselves to be using willpower to avoid watching the humorous video, and also believed that willpower was a depletable resource. It would be valuable to know whether performance improvements could be generated by simply manipulating beliefs. Exploring this in future research could have important implications for policies at the workplace and other related environments.

An important limitation of our study is that it assessed productivity on a novel task that required substantial concentration and allowed little room for error. In some work environments tasks are routine and may require little concentration or cognitive effort, and margins for error may be large. Connections between resource depletion and productivity in these sorts of environments are an open question worthy of continued exploration.

The findings of the present paper nevertheless seem to have practical implications for many work environments. An important one is that employers should not prohibit the Internet and yet leave it available. Instead, employers should either remove it entirely or, when doing this is impractical, allow employees a certain amount of time – maybe even as often as several minutes per hour – for personal Internet activity. Perhaps lunch-breaks can be somewhat shortened to accommodate “surf-time”. Alternatively, employers might consider allowing regular Internet breaks, in the same way that many currently accommodate short but not infrequent cigarette or coffee breaks. More generally, our study offers insights relevant for the design of efficient (productivity enhancing) policies directed towards providing employees breaks from regular work activity.

## Supporting Information

Text S1 **Experiment Instructions and Screenshots.**
(DOCX)

Figure S1 (TIF)

Figure S2 (TIF)

Figure S3 (TIF)

Figure S4 (TIF)

Figure S5 (TIF)

Figure S6 (TIF)
