# Supplementary material for: Temptation at Work
Source: PLoS One. 2013 Jan 30;8(1):e53713. doi: 10.1371/journal.pone.0053713 (PMC3559695; doi:10.1371/journal.pone.0053713)
Supplement: Text S1 — Experiment Instructions and Screenshots. (DOCX) [file pone.0053713.s001.docx]

**Temptation at Work**

**Alessandro Bucciol, Daniel Houser, Marco Piovesan**

**Supporting material: Experimental details**

The experiment was run at the Laboratory for Experimental Economics (LEE) of the University of Copenhagen. The LEE is a state-of-the-art facility devoted to research in experimental economics (more information here: <http://www.econ.ku.dk/cee/leelaboratory>). Before the experiment, subjects were randomly allocated to the two treatment groups (WT and NWT). The experimenter distributed a numbered card to each subject in each treatment group; each card gave access to a different cubicle in the same room, where to seat during the whole experiment. In total 61 subjects participated: 36 in WT and 25 in NWT. We decided to allocate more subjects in WT in case a fraction of them would have pressed the red button twice. However, only 1 subject eventually pressed the red button twice.

***The counting task (Phase 1 and Phase 3)***

In phases 1 and 3 we measure subjects' productivity through three and ten counting tasks, respectively. In each task subjects watch a video where eight individuals are passing each other one or more balls (of different colors). Subjects have to count the exact number of times a specific ball moves from one player to another one. When the video is over, subjects have to indicate their answer. The level of complexity varies from task to task with the number of ball passes subjects are asked to count. At the end of each counting task they receive a feedback with the correct answer, their guess and the points earned.

Notice that: i) each question is presented before the video starts; ii) the questions and videos change in each period but are identical for all subjects in a given period. Videos have a length comprised between 39 and 93 seconds, with an average of 60 seconds. The correct answers range from a minimum of 16 to a maximum of 69 ball passes. Therefore, the complexity of each task may vary due to the video length and the number of balls that are present at the same time in the video.

In phases 1 and 3 subjects earn points according to the precision of their answer. Subjects earn 100 points if they precisely report the correct answer, 65 points if the (absolute) difference between their guess and the correct answer is 1, 50 if the difference is 2 and 0 points if the difference is bigger than 2. At the end of the experiments points are converted in Danish crowns (DKK) according to the following exchange rate: 10 points = 1 DKK (1 DKK roughly corresponds to 0.18 USD).

***The temptation task (Phase 2 and Phase 3)***

In Phase two subjects in NWT watch a funny video (we used an episode of the Mister Bean series) for ten minutes, while subjects in WT are exposed to temptation. Temptation is represented by a big red button labeled "VIDEO". Subjects know that the video will start if they press the button, but they are asked not to do so. Subjects, however, hear the sound (mainly people laughing) from the loudspeakers of the laboratory. Therefore, subjects understand that the video is funny, but they cannot see what is going on and why people are laughing. This makes temptation strong. Moreover, subjects know that other people in the room have the possibility to watch this video and they are probably enjoying it.

If subjects in WT press the red button, a text message appears on their screen, warning that they should not have pressed the button. This is meant to recognize that pressure may be accidental. If subjects press the button one more time, pressure is no longer considered accidental, and the video appears.

All the subjects (both in NWT and WT groups) earn 250 points from this task, disregarding the pressure of the red button. This setting tries to mimic what happens every day in some workplaces. Workers are exposed to a large variety of temptations; employers may ask them to resist or they may let them succumb. Often there are no consequences if they do not follow employer’s directions.

Only one out of the 36 subjects in the WT group pressed the button twice and eventually watched the video. We disregard this observation from the analysis as we cannot use it to measure the effect of a prohibited temptation (in fact, the subject showed not to resist temptation).

**Instructions**

Welcome!

You are now taking part in an economic experiment which has been financed by various research foundations. During the experiment you can earn money. It is therefore important that you **read carefully the following instructions.**

Instructions are solely for your private information**. Do not communicate with other participants or look at the others’ screens during the experiment.** If you violate this rule, we will have to exclude you from the experiment and all the payments. If you have any questions, please raise your hand and **ask the experimenter or the assisting staff**.

During the experiment your earnings will be calculated in points. At the end of the experiment the points you earned will be converted into Danish crowns (*Danske kroner*) at the following rate:

**10 points = 1 DKK**

Your entire earnings will be paid in cash, privately, immediately after the experiment.

**The experiment**

The experiment consists of **3 phases** and will last **45 minutes** in total.

In the following page the rules of the three phases are explained.

**Phase 1**

In Phase 1 **three short videos** are about to be shown. In these videos there are some people passing each other a ball. You have to **count the exact number of passes**. At the end of the video, you have 10 seconds to **indicate in a box on the right the exact number of passes** you have seen.

The **number of points** you will earn depends on the **(absolute) difference between the correct answer and your guess** (see table below). For instance, if the correct answer was 10 and you have indicated 10 you will earn 100 points. If you have indicated 11, you will earn 65 points; similarly if you have indicated 9. If you have indicated 7 or 13, you will earn 0 points.

| **Correct answer – Your guess** | **Points** |
| --- | --- |
| 0 | 100 |
| + 1  - 1 | 65 |
| + 2  - 2 | 50 |
| + 3 (or greater)  - 3 (or greater) | 0 |

**If you are ready please press OK. You will be asked some control questions to check if the rules and payoff structure are clear.**

**Phase 2**

In Phase 2 you are randomly assigned to one of two groups, A or B. Subjects in group A will watch a funny video for few minutes. Subjects in group B will not watch the video but they will only hear the sound. At the beginning of Phase 2 you will be informed of whether you are in group A or B.

The video will start automatically only if you are in group A. If you are in **group B**, a red button labeled “VIDEO” will appear. **DO NOT PRESS THIS BUTTON**. If you are in group B and you press this button, you will receive a warning message. This will happen only the first time. If you press the button again the video will start.

At the end of Phase 2 you will earn 250 points.

**Phase 3**

Tasks in Phase 3 are identical to those in Phase 1. The only difference is that now you will see **ten short videos**. The payoff structure is the same as that of Phase 1.

**If you are ready please press OK.**

**Sequence of screenshots**

**A typical counting task in Phase 1 and 3**

**Figure S1.** Question

**
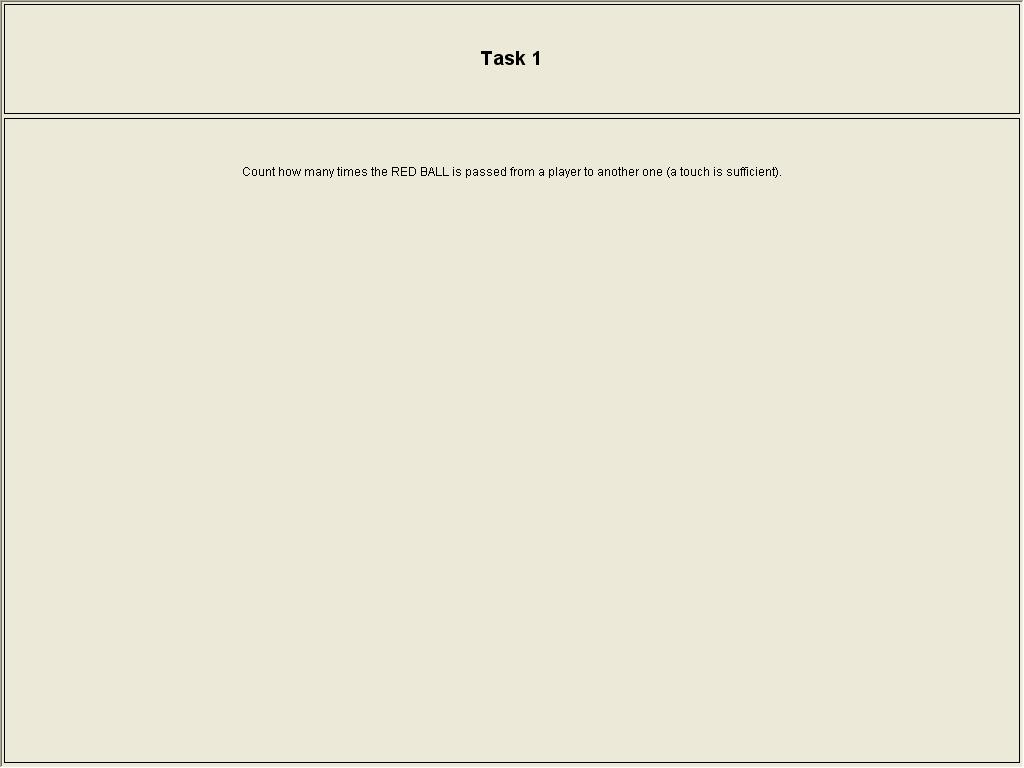
**

**Figure S2.** Answer

**
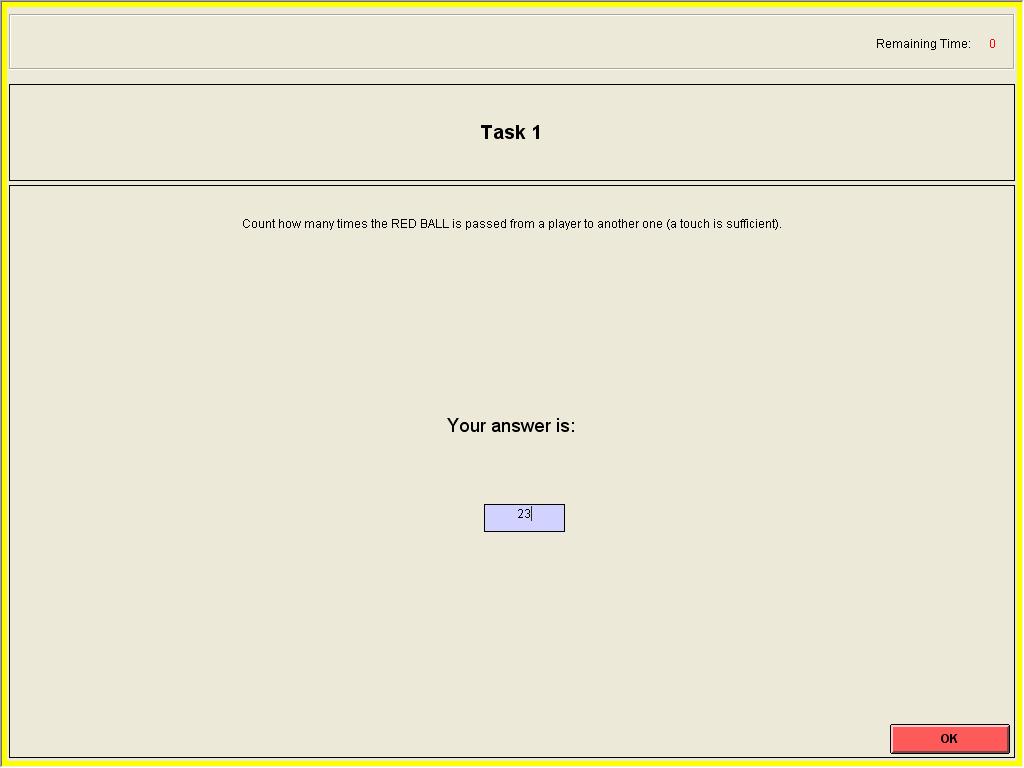
**

**Figure S3.** Feedback

**
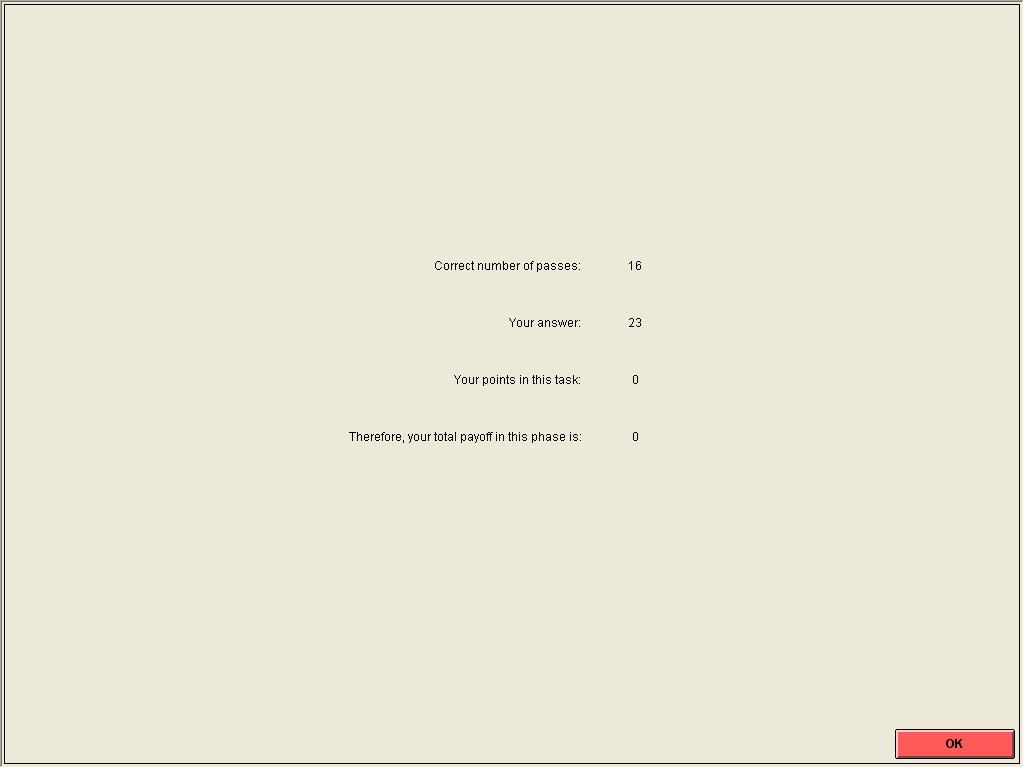
**

**Phase 2: No Willpower Treatment (NWT)**

**Figure S4.** Welcome screen

**
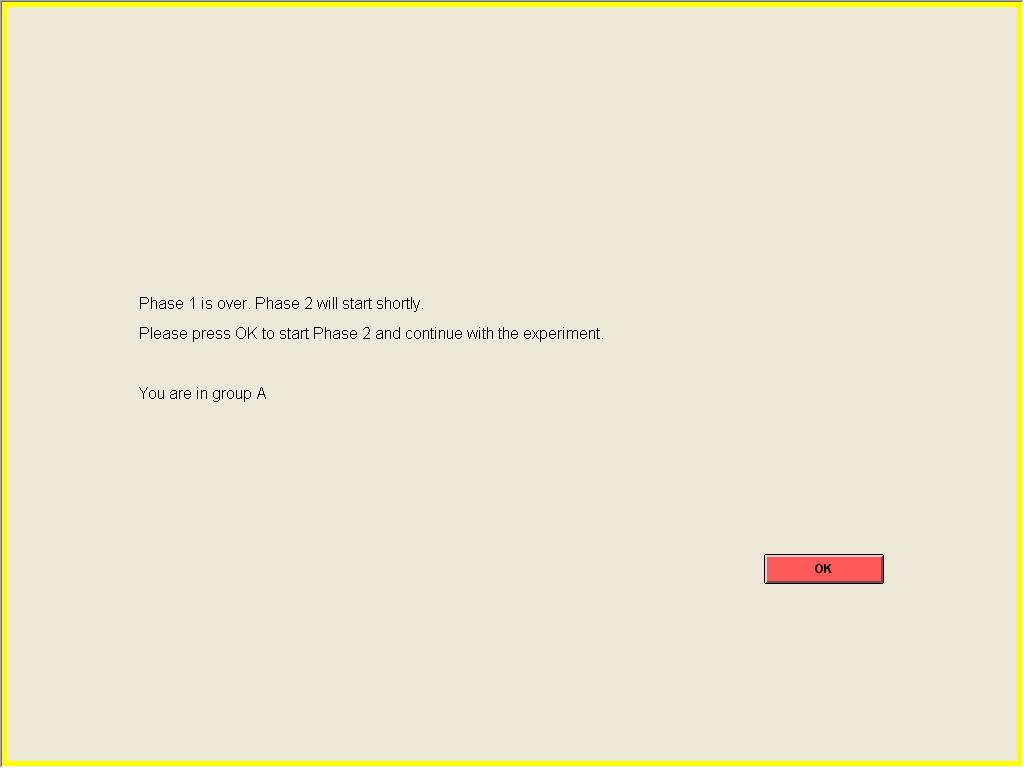
**

**Phase 2: Willpower Treatment (WT)**

**Figure S5.** Welcome screen

**
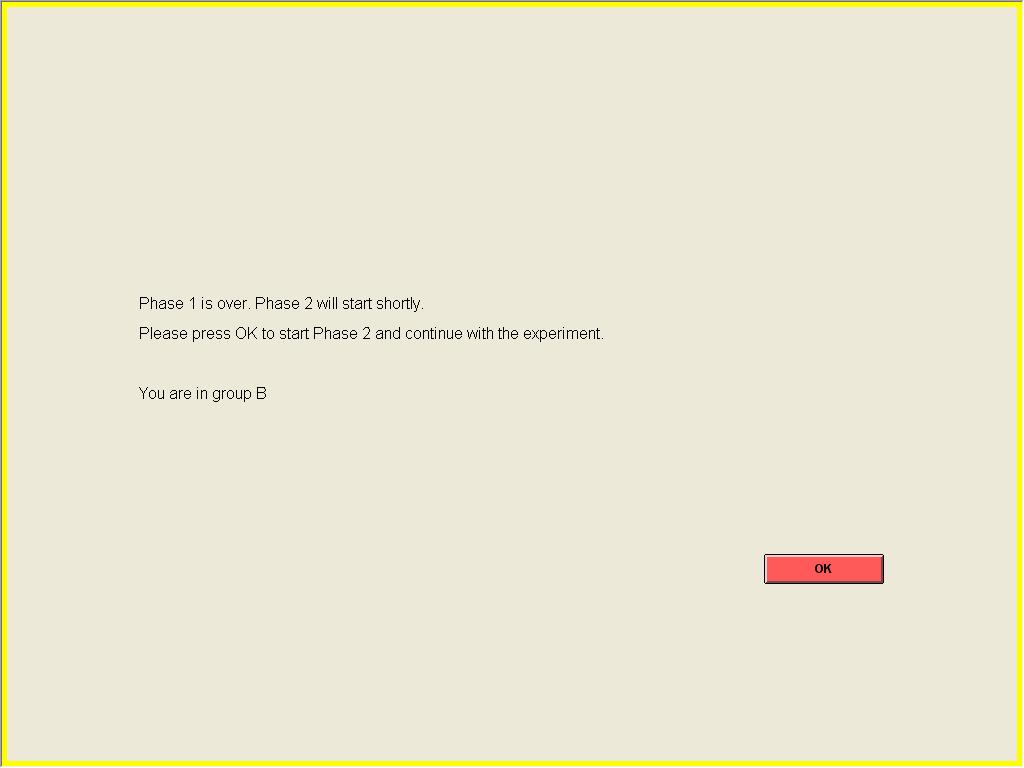
**

**Figure S6.** Temptation

**
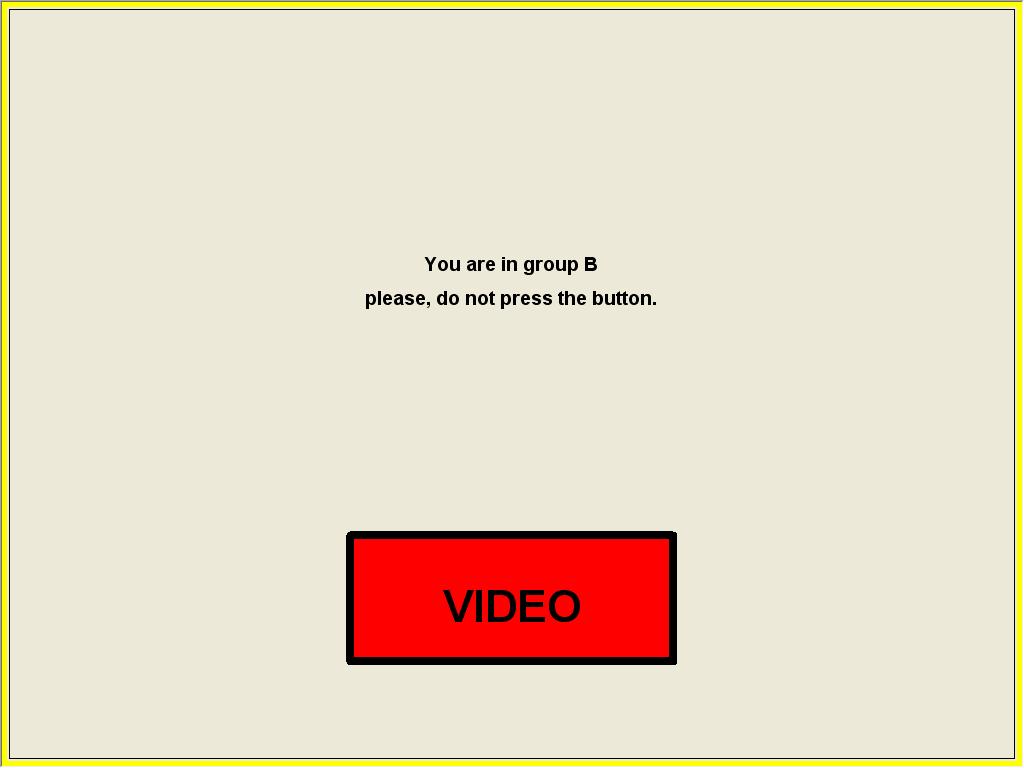
**
