# Supplementary figures and images for: Temptation at Work
Source: PLoS One. 2013 Jan 30;8(1):e53713. doi: 10.1371/journal.pone.0053713 (PMC3559695; doi:10.1371/journal.pone.0053713)

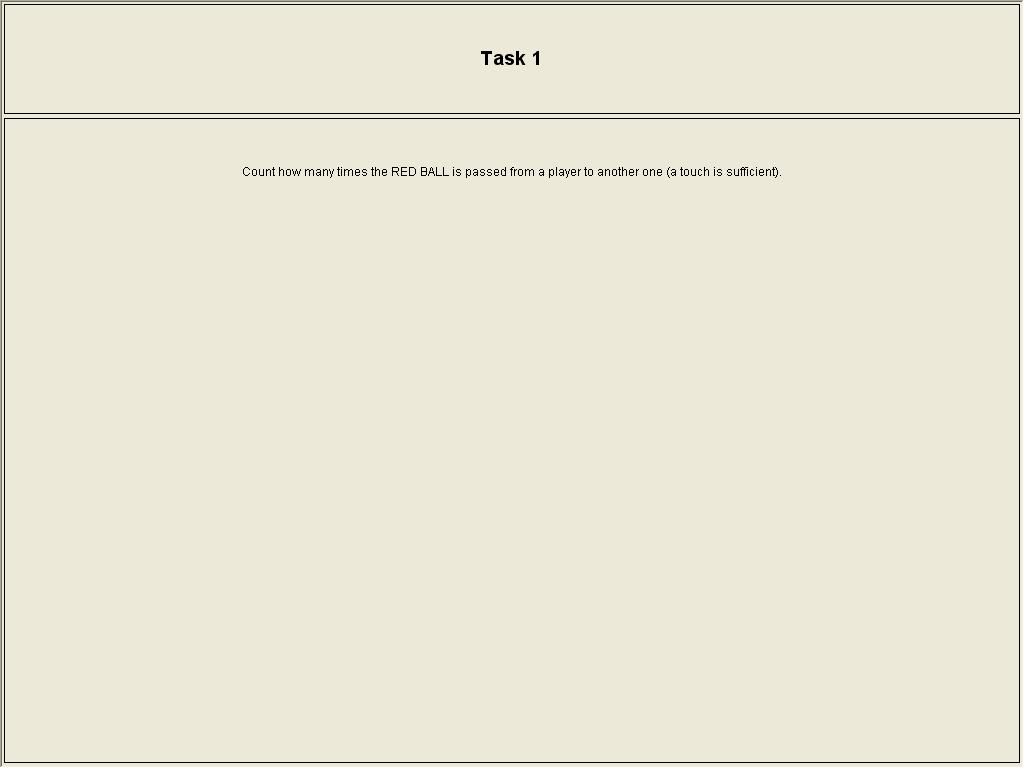

Supplement: Figure S1 — (TIF) [file pone.0053713.s002.tif]

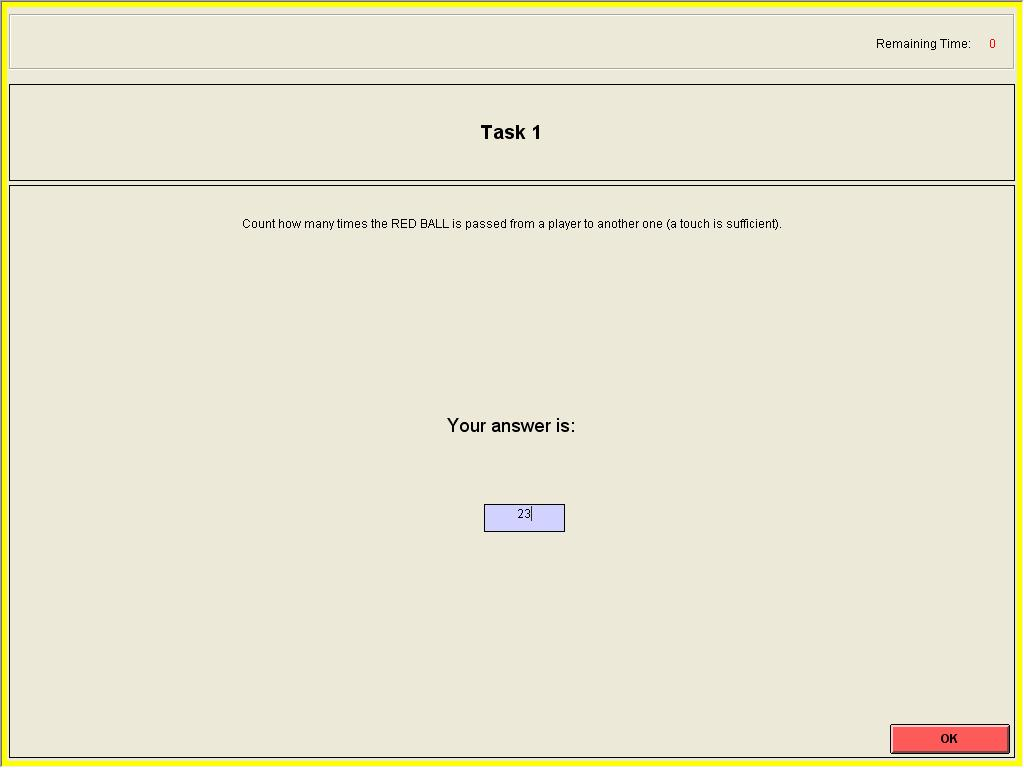

Supplement: Figure S2 — (TIF) [file pone.0053713.s003.tif]

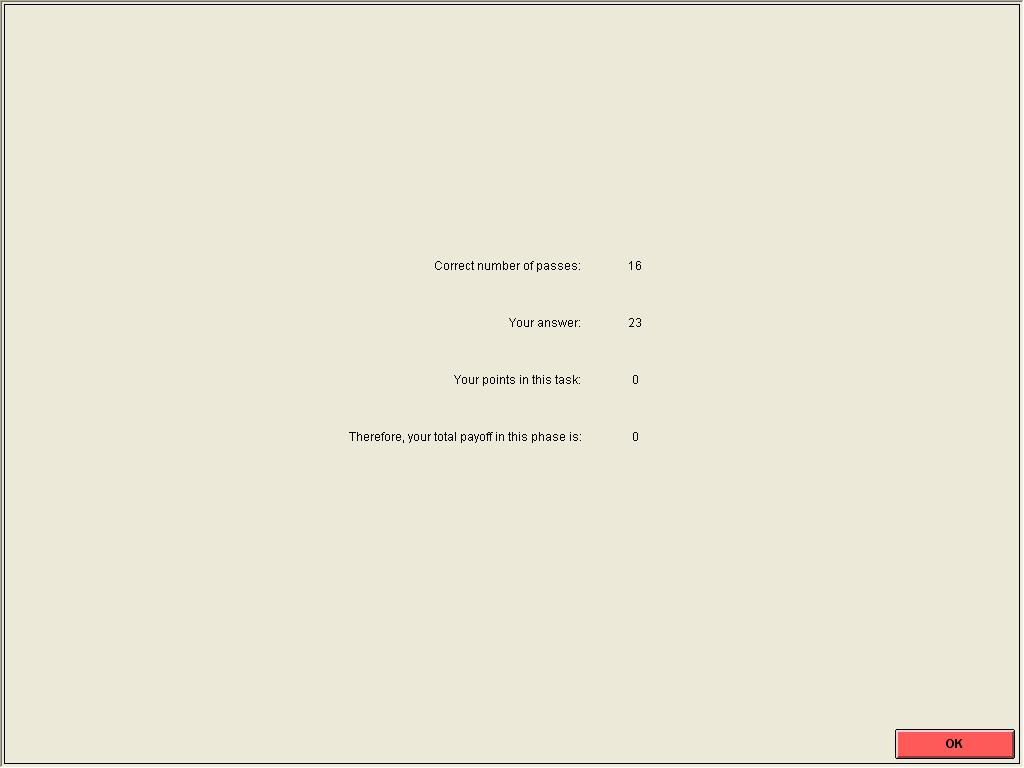

Supplement: Figure S3 — (TIF) [file pone.0053713.s004.tif]

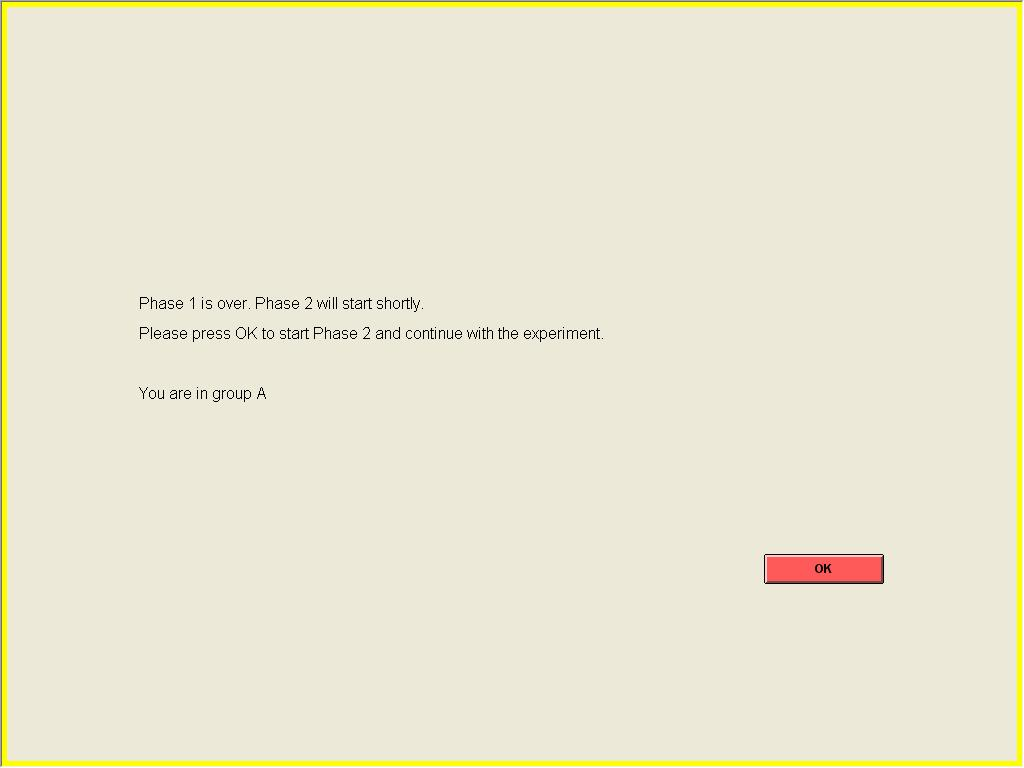

Supplement: Figure S4 — (TIF) [file pone.0053713.s005.tif]

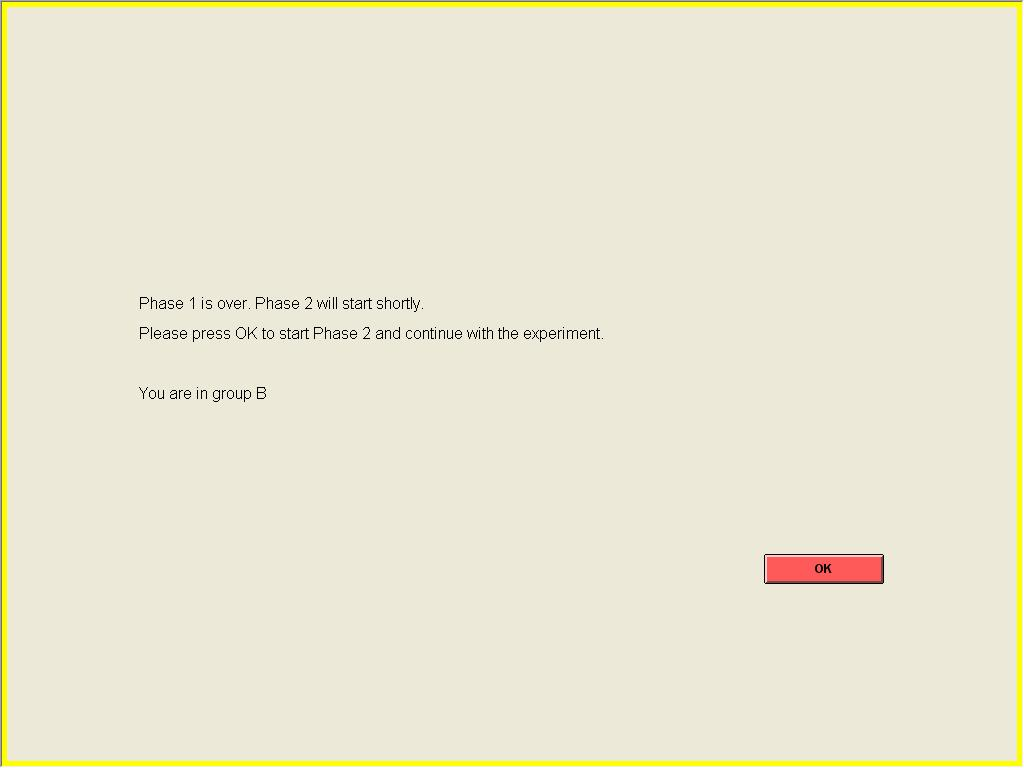

Supplement: Figure S5 — (TIF) [file pone.0053713.s006.tif]

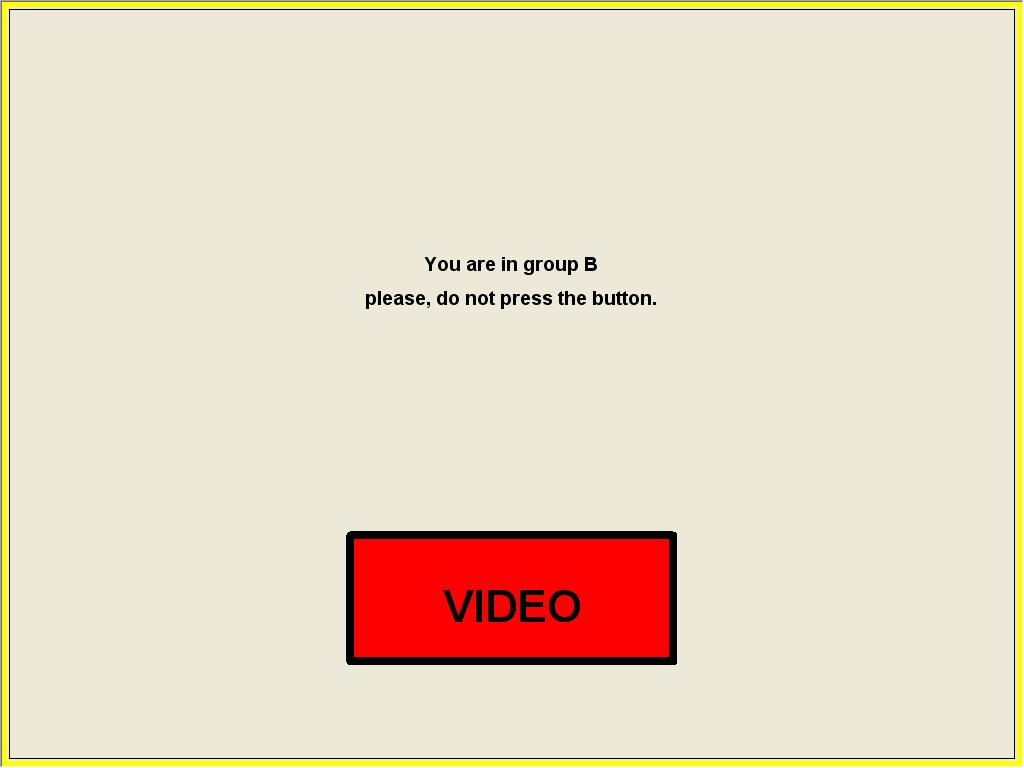

Supplement: Figure S6 — (TIF) [file pone.0053713.s007.tif]
